# “I Have Eight Different Files at Eight Different Places”: Perspectives of Youths and Their Family Caregivers on Transitioning from Pediatric to Adult Rehabilitation and Community Services

**DOI:** 10.3390/jcm12041693

**Published:** 2023-02-20

**Authors:** Kristina M. Kokorelias, Tin-Suet Joan Lee, Mark Bayley, Emily Seto, Alene Toulany, Michelle L. A. Nelson, Gina Dimitropoulos, Melanie Penner, Robert Simpson, Sarah E. P. Munce

**Affiliations:** 1 Department of Medicine, Sinai Health System and University Health Network, Toronto, ON, Canada; 2 KITE, Toronto Rehabilitation Institute-University Health Network, Toronto, ON M5G 2A2, Canada; 3 Department of Occupational Sciences and Occupational Therapy, Temetry Faculty of Medicine, University of Toronto, Toronto, ON M5S 1A8, Canada; 4 Division of Physical Medicine and Rehabilitation, University of Toronto, Toronto, ON, Canada; 5 Institute of Health Policy, Management and Evaluation, University of Toronto, Toronto, ON, Canada; 6 Center for Digital Therapeutics, Techna Institute, University Health Network, Toronto, ON, Canada; 7 Department of Adolescent Medicine, the Hospital for Sick Children, Toronto, ON, Canada; 8 Lunenfeld-Tanenbaum Research Institute, Sinai Health, Toronto, ON, Canada; 9 Faculty of Social Work, University of Calgary, Calgary, AB T2N 1N4, Canada; 10 Department of Pediatrics, Bloorview Research Institute, Holland Bloorview Kids Rehabilitation Hospital, University of Toronto, Toronto, ON, Canada; 11 St. John’s Rehab Research Program at Sunnybrook Research Institute, Sunnybrook Health Sciences Centre, North York, ON M2M 2G1, Canada; 12 Rehabilitation Sciences Institute, Temetry Faculty of Medicine, University of Toronto, Toronto, ON, Canada

**Keywords:** transitional care, youth, disabilities, rehabilitation, qualitative

## Abstract

Introduction: The number of young adults (youth) living with childhood-onset disabilities, and requiring transitional support to adult community and rehabilitation services, is increasing. We explored facilitators and barriers to accessing and sustaining community and rehabilitation services during the transition from pediatric to adult care. Methods: A qualitative descriptive study was conducted in Ontario, Canada. Data were collected through interviews with youth (*n* = 11) and family caregivers (*n* = 7). The data were coded and analyzed using thematic analysis. Results: Youth and caregivers face many types of transitions from pediatric to adult community and rehabilitation services, e.g., those related to education, living arrangements, and employment. This transition is marked by feelings of isolation. Supportive social networks, continuity of care (i.e., same care providers), and advocacy all contribute to positive experiences. Lack of knowledge about resources, changing parental involvement without preparation, and a lack of system responses to evolving needs were barriers to positive transitions. Financial circumstances were described as either a barrier or facilitator to service access. Conclusions: This study demonstrated that continuity of care, support from providers, and social networks all contribute markedly to the positive experience of transitioning from pediatric to adult services for individuals with childhood-onset disabilities and family caregivers. Future transitional interventions should incorporate these considerations.

## 1. Introduction

Many youth with childhood-acquired congenital or acquired physical or intellectual disabilities rely on rehabilitation services (i.e., rehabilitative healthcare services) and community services (i.e., services offered in the community to support social wellbeing and health) to help enable their community engagement and improve their wellbeing as they transition from pediatric to adult care [1,2]. Rehabilitation needs include vocational, physical, speech and language, occupational, and mental health rehabilitation [3]. Community supports generally include social services, such as recreation and employment services [4]. Family caregivers often play a key role in supporting youth in accessing the appropriate rehabilitation services and coordinating community care [5].

Youth living with disabilities are more likely than their non-disabled counterparts to engage with a high number of fragmented and complex health care systems during care transitions, leading to poor satisfaction with the care received [6]. Adult healthcare programs often have narrow eligibility criteria that are instigated by recent-onset disabilities or an indexed condition [7], making access to rehabilitation more difficult for adult individuals who acquired their disabilities in childhood [8]. Pediatric and adult rehabilitation practices can also differ vastly [9]. For example, multi-disciplinary clinicians have been found to be more readily available to youth and families in pediatric settings [10]. A lack of a community support system and continuity of care from pediatric to adult rehabilitation can lead to poor health outcomes as a result of lapses in treatment (e.g., functional decline) and decreased opportunities to participate in the community [11]. As such, there is a growing population of transition-age youth with disabilities and their family caregivers who are significantly underserved and report numerous unmet needs [12]. Rehabilitation professionals can play a critical role in facilitating transitions in care by connecting youth to adult services [13]. In this paper, we define a transition as “the purposeful, planned movement of adolescents and young adults with chronic physical and medical conditions from child-centered to adult-oriented health-care systems” [14] (p. 570).

Most of the existing research on the transition from pediatric to adult care has focused on healthcare and social care settings as a homogenous group of services [15], without a specific focus on community or rehabilitation services. As such, the facilitators and barriers to implementing and sustaining both of these services during the transition from pediatric to adult care are not well established. An understanding of such factors may help identify strategies to promote continuous rehabilitation *and* community care and improve experiences with health services and the quality of life for youth with disabilities and their family caregivers. Thus, the purpose of the current study was to explore experiences with transition from pediatric to adult rehabilitation and community services among youth with childhood-acquired disabilities and their family caregivers, as well as perceived facilitators and barriers to accessing and sustaining both rehabilitation and community services during such transitions.

## 2. Methods and Materials

### 2.1. Study Design

An exploratory, qualitative descriptive study grounded in naturalistic inquiry was conducted [16]. Ethical approval was sought from The University Health Network (#22-5023.0). The Consolidated Criteria for Reporting Qualitative Research (COREQ) was used in the reporting of our methods [17].

### 2.2. Setting

This study occurred in Toronto, Ontario, Canada. In this jurisdiction, Ontarians have access to publicly funded hospital and physician care, including ‘medically necessary’ rehabilitation. Variation occurs across the province regarding access to rehabilitation (e.g., variation in resources, service availability) [18]. Transitions from pediatric to adult rehabilitation and community services often occur at or before 18 years of age [19]. In addition, community services are often restricted to either pediatric or adult service offerings [20].

### 2.3. Participants

Participants included youth and family caregivers (e.g., family, friends). To be included in the study, youth were required to be between 19 and 30 years of age, English speakers, able to provide informed consent and living with a childhood onset disability. Family caregivers were required to self-identify as the main care provider (i.e., providing care or managing care processes) to a youth between 19 and 30 years who is living with a childhood disability, and is English speaking. The first author screened all participants for eligibility during a telephone screening. A total of 14 youth participants and 7 caregivers were screened.

### 2.4. Recruitment

Participant recruitment involved recruitment from a local hospital center and a local community service agency that provides services to youth and adults with childhood-onset disabilities (i.e., a physical or mental impairment developed during childhood). Eligible patients and family caregivers were identified by providers via the LIFEspan Service and their emails were provided to the research team to contact. In addition, the research team used social media to recruit participants. Purposive sampling strategies were used to recruit participants with a broad range of perspectives (e.g., asking providers for caregivers, sending social media messages seeking youth living with various forms of disability) [21]. Interested participants contacted the research coordinator, who explained the nature of the study and provided participants with an opportunity to have their questions answered before seeking verbal consent. After verbal consent was obtained, participants provided written consent, and a phone interview was scheduled at a mutually convenient time. None of the participants were previously known to the research team.

### 2.5. Data Collection

Two female qualitatively trained researchers ([Blinded for review]) conducted the in-depth interviews using a semi-structured interview guide (see Table 1 for Sample Interview Questions). Each participant was interviewed once. All interviews were audio-recorded, professionally transcribed verbatim, checked for accuracy by comparing the audio to the transcription, and all identifiable information was removed. Immediately after the interview, participants were offered the opportunity to debrief, discuss their experience of the interview, and ask any questions to the interviewer. The interviewer completed notes immediately following each interview to help reflect on the interview that just occurred and note areas to probe during subsequent interviews. Reflexive notes helped the research team begin to consider thematic saturation as well as any researcher bias [22]. Data collection ceased when thematic saturation was achieved (i.e., the point where future interviews were believed to not reveal new findings, while recognizing “saturation” may not be fully realized until the end of data analysis). Our efforts for determining saturation were supported by our technique of collection and analyzing data simultaneously [23]. 

### 2.6. Data Analysis

Data analysis occurred concurrently with data collection. To ensure a rigorous thematic analysis, the components of Braun and Clarke’s six-phase process were followed [24]. Firstly, the first author reviewed all transcripts by reading them over several times to facilitate familiarization with the data and not initial ideas. A subset of the transcripts was reviewed by the other members of the research team in the same fashion. Next, the research team met to discuss initial ideas, key concepts, and perceptions to help inform a preliminary understanding of the data. Eight transcripts, including transcripts from both youth and family caregiver interviews, were then reviewed using open line-by-line coding of the data by the first two authors ([Blinded for review]) and the senior author ([Blinded for review]). This coding involved analytic codes that aligned with the study’s aims. The authors then met weekly to discuss the coding process and gain a holistic understanding of participants’ experiences. The key concepts informed the development of a final codebook of inductive codes (see Table 2 for a sample of the codebook), which the first author applied to all the transcripts. The second author coded a subset of the transcripts (50%) using the same codebook. Similarities and differences in coding were compared. NVivo 12 helped to organize the data and facilitate the coding process [25]. The coded data were compared within and across the transcripts and discussed during the weekly research meetings. Once all data were coded, several team meetings occurred where codes were grouped together with other similar codes, in order to identify potential thematic categories [24]. During this process, codes were compared across participant types (i.e., youth or family caregiver) to compare and contrast findings and identify core patterns and relationships within the data [24]. Preliminary themes were continually reviewed, and a thematic map was constructed to describe the relationship between themes [26]. This thematic map was reviewed by the entire research team until themes that were considered final were conceptualized. These final themes were then reviewed against all the coded data to ensure they reflected the shared meanings across the data. This step was done by three authors (Blinded for review). The themes were considered final when they appropriately reflected data from all participants, were supported by verbatim quotes, and a consensus was reached amongst the entire research team about their content, which provided a rich and detailed description of the data [24]. The final themes were then named accordingly, and participant quotes that represented each theme were selected [27]. Member-checking (also known as reliability checks) was not conducted with participants (e.g., participants did not review the final themes) [28,29].

## 3. Results

The study included 18 participants, comprising 11 youth living with childhood acquired disabilities and 7 family caregivers (see Table 3 for Participant Characteristics). From the 18 participants, 4 individuals were interviewed as dyads (i.e., two pairs of related family caregivers and youth interviewed together). Interviews were 30 minutes to 75 min in length (average 60 min). All but one family caregiver was the biological parent of a youth living with a childhood acquired disability. The remaining family caregiver participant was a close relative. Characteristics of the caregivers and youth are reported in Table 2. In the results sections below, we use quotations to illustrate the various themes. Quotations are marked by the participant type (i.e., caregiver [CG] or youth [Y]), participant ID number, and sex. We also indicate the youth participant’s primary diagnosis.

### 3.1. Experiences during the Transition to Adult Care

#### 3.1.1. Losses, Grief, and Feelings of Isolation

Participants described transitioning as being an isolating experience. Youth frequently shared that they did not know peers living with disabilities and, thus, found themselves unable to relate to their peers who were not living with a childhood-onset disability as they were not experiencing the same transitions. For others, social isolation and loneliness arose from having no close friends to support them during transitions, despite making efforts to build relationships. As one participant shared, “*it’s great when you’re receiving support groups and you’re around kids your age, then you grow up and you go to school and you try, but no one knows what else you’re going through at the same time.*” (Y13, female, intellectual disability). Some participants shared moments of their experiences when they felt that other individuals without a childhood-onset disability did not genuinely understand them and their situation, such as their classmates and other networks of friends.

Moreover, youth described that in the absence of attending their previous pediatric community services and support groups, they felt a sense of loss from their previous activities and routines. As one participant shared, *“Like before you get up, you go to a group once a week, you see people who have known you since you were 5. Then you lose this”* (Y13, female, intellectual disability). Participants frequently referred to the loss of their pediatric rehabilitation providers and peers in group activities.

#### 3.1.2. Multiple, Simultaneous Transitions

Participants described that the transition from pediatric rehabilitation and other services often occurred concurrently with other life transitions, such as those related to education (e.g., secondary to post-secondary), living arrangements, and employment. Family caregiver participants described either needing to support the youth through these transitions or mourning that the youth in their care may not reach these normative milestones. Nonetheless, these milestones seemed to also mark an understanding of the need to transition from pediatric services to adult services for both youth and family caregivers. One caregiver participant shared, *“when you go from high school to university it’s a huge step […] It’s a whole other phase of life. You move away. You start over. You have to help them find services all over for these needs.*” (CG5, female).

Regardless of the context and transitions that were occurring, all participants described facing multiple transitions at the same time as an overwhelming experience. Many participants associated these feelings of being overwhelmed with uncertainty in the future and a lack of preparedness (e.g., not knowing what would be expected of them). In some cases, this meant that youth or family caregivers had to prioritize accessing adult-appropriate rehabilitation and community services rather than preparing for other life transitions such as graduating high school or dating. When asked as to why rehabilitation and subsequent community services were prioritized, participants largely described that these services would need to be in place to facilitate the other transitions. One participant described *“I had to go there [Adult Rehabilitation Centre] or else I feel like I couldn’t go to school or do other things, but I didn’t like being there without people I was comfortable with.*” (Y1, female, intellectual disability).

### 3.2. Barrier and Facilitators to Undertaking and Sustaining Adult Community and Rehabilitation Services

#### 3.2.1. Barriers

##### Lack of Knowledge about Navigating and Accessing Adult Services and Resources

Participants described their limited understanding of adult rehabilitation and local community services. Consequently, participants expressed difficulty in obtaining timely, accurate information about currently available services and their eligibility requirements. Some youth participants shared that they knew nothing about organizing their own service needs when they first began their transition to adult care (e.g., how to call to book appointments with a specialist, managing medication). Similarly, family caregivers assumed that they would receive more guidance from those in pediatric services but could not find professional guidance. One family caregiver shared, *“I found that there really isn’t a transition still, we’re trying to pick up the pieces, so to speak. We can’t find out who to ask.*” (CG5, female).

In the absence of informational support and knowledge of service availability, participants described facing lapses in their rehabilitation treatment or not enrolling in community services they would have benefited from, such as support to help them find employment. One participant described, *“I’ve had friends, same program, where they’ll need more care, and it’s a massive drop off for them. Then they’re kind of scattered, and they settle in new … what’s the word … they’re kind of lost in the first few months of [adult rehabilitation), kind of lost. “* (Y14, male, acquired brain injury).

Some participants described significant difficulties in understanding service directories (e.g., City Services for People with Disabilities) and thus struggled with the variety of information available online. One participant shared, *“You can read that internet screen, but you have to be careful what you read there. It’s not always up-to-date stuff”* (Y2, male, cerebral palsy). The difficulties were further exacerbated by communication challenges, particularly for non-native English speakers. Similarly, participants did not have a clear idea about how to interpret eligibility criteria for services.

*“I think another thing too that makes it difficult is my parents are immigrants. English is not their first language so I think just navigating all of that as well was just an extra barrier for them and for me to understand stuff. [Once] my mom turned to me and went like, “oh, I think you actually would have qualified for a certain thing, but we just didn’t know to apply for them because we just didn’t know about them” [..] I think that was I guess a struggle that we faced*.

(Y6, male, acquired brain injury)

##### Sudden, Increased Accountability with Concurrent Decrease in Parental Support

The transition to adult rehabilitation and community services was marked by a stark increase in youths’ responsibility over their care. Youth participants frequently described how, during their childhood, they were largely dependent on their parents for organizing their rehabilitation and/or registering them for the appropriate social support service. As youth transitioned to adult-appropriate care and services, their parents’ involvement lessened, possibly due to parental (realistic or unrealistic) expectations of independence or a change in living arrangements. While some youth appreciated the new responsibility, the majority described frustration and a lack of preparation.

One youth participant described, *“I was getting the emails and [my mom was no longer receiving them] and I just didn’t know what to even do, so I may have even missed out on stuff. That’s frustrating that they don’t communicate with you, then you turn 18 and they do. […] they [service providers] just assume your parent isn’t involved.*” (Y10, female, cerebral palsy).

Another participant shared, “*usually I think at [*pediatric rehabilitation center*] you’ll see is Name-X or Name-X, the parents are always the ones doing all the talking and everything, they’re always supposed to do everything. When you’re 18, you’re like, [explicit language], I’ve got to do this kind of different thing, it’s all of a sudden*” (Y14, male, acquired brain injury).

Many youths still involved their parents when trying to access care by asking them for advice, given the parents’ knowledge and understanding of organizing services. However, youth described that they had to do so on their own time and outside of scheduled appointments or conversations with healthcare providers. One participant said, *“I’m really lucky because my parents have been good at researching and finding new ways to help me improve my skills.*” (Y8, female, cerebral palsy).

In some contexts, youth were no longer living with their families or described a decline in parental involvement, and this resulted in youth exhibiting signs of frustration or a lack of motivation to find services to meet their needs. Some youth described wishing that their parents could attend appointments, renew prescriptions, or communicate with rehabilitation professionals with questions.

Family caregivers described the challenges of wanting to provide their youth with autonomy over their care but still needing to assist them in making decisions. For example, family caregivers of youth with non-verbal status described the challenge of needing to be involved in care and service planning, such as scheduling and attending appointments, but often having to justify to service providers why they had to be involved. The lack of involvement in care planning or information about youth’s service needs caused further burden on family caregivers, and it led to feelings of a *“lack of control and powerlessness*” (C1, female).

##### Lack of System Response to Evolving Needs

As youth and families transitioned from pediatric to adult services, they also described a shift in their needs. Youth described being faced with an increasing need for physical rehabilitation as well as occupational support. Many of the youth participants described their level of functioning as having plateaued and/or learning to live with their current physical functioning. Youth frequently described how the pediatric rehabilitation they received assisted them in adapting their functioning, but now they desired supports that were related to their nonmedical needs. For youth, these supports were related to job-seeking, post-secondary education, transportation, and socialization. Many youth participants believed that non-physical forms of rehabilitation, such as occupational support (i.e., to support job-seeking), did not exist. In some situations, this resulted in not accessing rehabilitation altogether and frustration experienced by both youth and family caregivers:

*“Previously I was looking for employment services which they couldn’t really offer me because all they focus on is physical care so I have that now so that would be what I was looking for that they didn’t give me. And as far as other services I can’t think of any right now other than employment services that are important to what I’m going through right now.”*

(Y4, male, cerebral palsy)

#### 3.2.2. Facilitators

##### Social Networks Support Positive Coping

Participant narratives also highlighted numerous examples of tangible (i.e., hands-on) support that they received from their social networks. Even though all the interviewed family caregivers reported being the primary source of support for their youth, they did mention occasionally receiving tangible support from others, such as assistance with physical transfers or housekeeping. Youth described support such as assistance with transportation to services, physical transfers from wheelchairs, and mulling over ideas around what services to select. Emotional and tangible support contributed to alleviating feelings of burden associated with transitions and made the transitions feel more manageable. One youth simply stated, *“just being able to talk things through helped me cope, that was very helpful to me”* (Y5, female, intellectual disability).

##### Advocacy Skills for Both Youth and Family Caregivers

Youth and family participants indicated that advocacy skills were essential to securing community and rehabilitation services in adulthood. Participants described that, in the absence of information and sometimes a lack of publicly funded rehabilitation, they had to coordinate information from various professionals and service agencies. Participants described that the structure of rehabilitation and community services required some knowledge about overcoming barriers to eligibility criteria, and thus, youth and their family caregivers were required to advocate for their need for services and their fit with eligibility criteria. One participant described their need to advocate as “*they [service providers] won’t give you anything unless you’re pretty adamant and tell them what you want. You have to prove you fit their boxes.” (Y2, male, cerebral palsy).* Another participant shared, “*advocacy is the biggest thing [to accessing support] and you have to seek it out yourself and you’ve got to advocate and push for it and sometimes you get fortunate*” (CG5, female).

Youth participants described how advocating for services during care transitions resulted in them becoming resilient and perseverant. Similarly, family caregivers described the need to be vigilant over service providers, to ensure that they were following through with the services that the youth was promised to receive, as well as vigilant that the youth had their needs met by the service provider given a lack of specialized care. Both youth and family caregiver participants described that advocacy efforts required significant investments of their time and emotional energy.

##### Continuity of Care from Adult Service Providers

From the participants’ perspective, organizations offering care, such as rehabilitation, should be merged with pediatric organizations and be more collaborative. This would result in more easily accessible and continued services for when youth with disabilities become adults. Along the same lines, participants shared that they desired a holistic care system for youth living with disabilities that involved collaboration between health and social care services to address their multidimensional goals and needs (i.e., not just medical) and that continuity of care would involve ongoing communication with pediatric providers prior to the transition. In the absence of such a coordinated system, patients have had to locate and integrate information and resources themselves.

*“My family physician isn’t necessarily well versed in disability and mobility issues and all those types of things and the way that my disability could potentially impact health aspects in my life […] I see a gynecologist and a naturopath and things like that, and they don’t really speak to the other doctors in my life, so I kind of just mediate whatever they say and then I kind of take all three of their advice and figure out what’s best for me […] I have eight different files at eight different places.*”

(Y8, female, cerebral palsy)

Moreover, participants also believed that continuity in services was compromised by the need to be referred to these services, and oftentimes, those working in pediatric health and social care did not know of the services available for adults. One participant shared, “*If there could be some kind of continuity, that would help the individual and the family in general instead of, okay start all over again. Reapply. Find out how to be referred.*” (C5, female).

Youth who had received care from an adult care provider, such as a family doctor, instead of a pediatrician as children reported having easier transitions to adult rehabilitation as their adult care provider facilitated those connections to adult services. Moreover, adult rehabilitation clinicians had the existing disease and treatment knowledge necessary to make recommendations for services, which resulted in individualized intervention plans for youth. Youth described that receiving rehabilitation from a service provider who already knew their abilities and diagnosis provided continuity in the monitoring of their functioning, even if they received new services (e.g., vocational therapy). As a result, seamless care was provided which cultivated a sense of being known and well cared for. These processes helped youth feel a sense of trust in the adult care system, as they felt they were in the same group. Similarly, family caregiver participants felt a sense of relief that there was at least one familiar contact for the youth who had known them since childhood. Family caregivers noted that they had to constantly initiate and maintain partnerships with their own care provider to be able to enroll their family member in their care. Many youth and family caregivers described how having a general (i.e., not pediatric) adult rehabilitation care provider during childhood allowed for the transition process to begin earlier. One participant explained, *“That is why I’m glad I saw an adult rehab person at 15 not 18. I was able to just continue on with most of the normal rehabilitation professionals I saw” (*Y9, female, cerebral palsy*)* Moreover, participants reported a preference to receive care from existing care providers as a result of familiarity.

#### 3.2.3. Barrier or Facilitators Depending on Circumstances

##### Finances

Some participants offered examples of the funding context that supported them in obtaining services. For youth and family caregivers with the financial resources to do so, access to privatized (i.e., out-of-pocket, non-government healthcare plan funded) rehabilitation services helped ensure the sustainability of care following pediatric care. However, many family caregivers described the high cost of caring for a youth with childhood acquired disabilities, which was largely related to the cost of specialized equipment that was often not covered by public funds. Some family caregivers identified the high cost of these additional expenses as the reason that they had not accessed other services such as respite or private rehabilitation.

*“So, if you’re going to attend that kind of program for the rest of life, it isn’t possible, who can afford that, $200.00 a day. Even more. So, I’m saying, even out there, rehab program-wise, they do have programs, but you have to pay or you wait. I don’t see choices.*”

(CG2, female)

Almost all participants noted that there was a perceived inequitable funding disparity between pediatric and adult services that hindered their ability to access services. For example, participants, especially family caregivers, believed that youth rehabilitation and community services received more funding from the government than adult services. Moreover, access to free or low-cost services often came with very strict eligibility criteria (i.e., by age, medical diagnosis, living arrangement) preventing many participants from accessing them, or were unsuitable for youth with particular needs (e.g., the need to receive care in home). As such, the stability of continuing to receive rehabilitation or accessing community care was threatened.

*“A lot of these services for young adults are not covered, if you want to go that route you have got to pay for them yourself. There are the ones that are covered through the government and there are either huge waiting lists or again, as I said, they’re more for severe cases. In pediatrics, every young child is covered.”*

(CG5, female)

## 4. Discussion

The current study sought to explore youth living with childhood acquired disabilities and their family caregivers’ experiences with the transition from pediatric to adult community and rehabilitation services. Youth and family caregivers expressed a great desire for continuous and collaborative care across pediatric and adult services. Several factors helped youth and family members access services including, but not limited to, social support and the development of advocacy skills. Conversely, factors that have hindered successful transitions included lack of knowledge of the available rehabilitation and community resources, the sudden increased accountability of youth in overseeing their own care, and the lack of system responsivity to the evolving physical and social support needs of youth. Depending on the socio-economic circumstances of the individual involved, financial considerations could be a facilitator, facilitating access to private services, or a barrier to care, where high costs make private care unobtainable to those less affluent. Collectively, these findings highlight an intersectionality of personal, social, and cultural factors that can serve to accentuate disability at a time of increased need for support.

Existing literature suggests that receiving information about service availability from healthcare providers, such as family physicians, is important as providers are perceived as having knowledge about the conditions and needs of individuals with complex care needs [30]. Our results suggest that youth connected with primary care providers prior to reaching adulthood have more seamless transitions and are able to access adult services more easily. The degree to which continuity in care helps to mitigate feelings of isolation, improve information sharing, and manage expectations warrants further exploration. However, we found that continuing to receive rehabilitation from someone familiar with the youth’s abilities helped foster trust that the adult services they will receive will be supportive of their specific/unique needs. Opinion leaders have argued for the shared responsibility of pediatric and adult care clinicians [31]. We argue for broader sector partnerships between pediatric and adult care providers and between rehabilitation and community care providers, such that care coordination is not limited to the physical rehabilitation sector to help expand access to comprehensive services. For example, adopting policies that allow and support healthcare providers to work across acute, rehabilitative, and community care sectors may help facilitate such partnerships [32].

Participants in our study highlighted a shift of responsibility from parent to young adult with regards to accessing services. Our results highlight the inherent variability in readiness and desire of youth to undertake this responsibility in accessing rehabilitation and community services at times of transition. While the shift in responsibility and involvement from parents to youth is well supported in the literature and existing models of care and transition interventions [12,33], the nature of youth involvement should be individualized and the diversity of patient and family needs and abilities should be considered. As parents or surrogates are essential in encouraging the development of the skills needed to be independent and autonomous [34], providing strategic opportunities to help support parents and other caregivers in facilitating youth to be advocates appears necessary. Further research exploring how youth and family caregivers develop advocacy skills could inform the development of such a support program. In addition, focusing on removing communication barriers by developing culturally-sensitive mechanisms to support transitions is warranted [35]. Moreover, the timing of the transition should also consider individual circumstances. Individual circumstances for consideration include the degree to which youth wish for their parents to be involved, family caregivers’ perspectives of the need to be involved, and the ability of adult service providers to meet the holistic needs of youth [36]. Future studies should describe the characteristics of families so that transition programs can be developed that are flexible enough to address unique family circumstances and help with changes in responsibilities [37]. Moreover, service agencies should not assume a complete shift in autonomous decision-making for youth (i.e., assume no parental responsibility or need for support). Lastly, emotional support strategies to decrease transition-related frustration seem very important to youth with disabilities who are already overwhelmed by multiple transitions occurring in their lives [38].

Findings from our study suggest that transitional services should be flexible and responsive to meet the varied needs of youth, including being able to address vocational, educational, and transportation needs. Our study suggests that it would be beneficial to introduce the transition prior to the age of adulthood to help facilitate continuity of care and a sense of preparedness in youth and their families. As a way of facilitating positive transitions early, transitional programs should have youth engage in transition practices such as learning advocacy skills and what to expect to help prepare them before they turn 18 [39]. At the same time, such programs must consider the role of parental involvement and the active role parents want to have in the youth’s care. As such, interventions should consider the unique needs of family members, including the need for them to be educated on any evolving healthcare needs or progressions in illness [40,41]. Future interventions should also consider how to best connect youth and their family members to peers with similar lived experiences and professionals trained to address social aspects of health to serve as social and emotional support [42]. Patient navigation is one example of a model of integrated care that is now increasingly emerging in a range of healthcare settings for individuals with chronic needs [43] and is a potential solution to meet these needs. Delivered by trained peers or healthcare professionals, patient navigation can help facilitate care coordination activities across transitions in care settings by helping patients and their families overcome perceived barriers to care through advocacy, case management, information sharing, and support [44]. While patient navigation programs may be a viable option to support youth and families transitioning from pediatric community and rehabilitation services, it is important to understand the ideal components of a patient navigation program to meet their needs. Few studies have explored the experiences of youth and families who use patient navigation services, particularly when the focus of the program is on transitions from pediatric to adult rehabilitation and community services [45]. As such, future work is encouraged in the area of patient navigation for youth with childhood acquired neurodevelopmental disabilities as they transition from pediatric to youth services. Specifically, a better understanding of the ideal patient navigation program [46], the barriers and facilitators to implementing such programs [43], and their outcomes is warranted.

### Limitations

There are a few limitations to this study that should be considered. While Ontario, Canada, as a whole, was considered suitable as the jurisdiction of this research given its vast variation in care delivery and geography [47], the majority of our participants lived in, and received care in urban settings. Moreover, all participants were English speakers and did not have any detectable communication difficulties. Some participants identified as being non-native English speakers during the interviews. This may have influenced the information gleaned from our interviews and our interpretation/analysis of that information. Future studies are encouraged to consider a multilingual approach to data collection. Despite this, participants’ experiences may reflect the context of a multicultural urban healthcare environment and, thus, might be useful to inform transitional care service delivery in other urban settings. Moreover, while the quality of phone interviews has been supported in the context of qualitative research [48] and the interviews generated rich discussions, the study analysis could not involve non-verbal data or non-verbal participants. Participants in this study were predominantly Caucasian and Asian, and therefore, they likely reflect most closely the perceptions of people from these ethnic backgrounds. Lastly, this study did not investigate the perspective of healthcare providers and other professionals who work in social-rehabilitation settings. Interviewing these providers may provide additional insights into the availability and experiences of support during transitioning from pediatric to adult rehabilitation and community services.

## 5. Conclusions

The transition from pediatric to adult rehabilitation and community services can be a challenging experience for youth living with childhood-acquired disabilities and their family caregivers. The experience of social support and the application of advocacy skills were identified as key factors that contributed positively to this experience. On the other hand, a lack of knowledge about services, increased responsibility thrust on youth regarding their own care when they are unprepared to do so, and a lack of services for non-medical needs were barriers to accessing community and rehabilitation services. Financial resources could be a barrier or a facilitator to sustaining care throughout this transition. Understanding how these factors can be leveraged to prepare youth and their families for transition is critical. Future research is warranted to develop and evaluate interventions and programs to improve readiness for transitions, which will help increase knowledge of available resources, help support youth to be more accountable for their service provision, and potentially reduce feelings of isolation caused by changes in care.

## Figures and Tables

**Table 1:** Sample Interview Questions.

Sample Question	Probes
Could you describe for me your/your family member’s experiences of transitioning or moving from the pediatric health care system to the adult health care system?	How prepared did you/your family member feel in the beginning? How do you/they feel now? What were the positive aspects of this experience? What were the negative aspects of this experience?
Looking back, what else do you think could have been done (support, training, resources, programs, services, etc.) to make this process easier for you/your family member (and perhaps could still be done)?	What or who helped make this process easier for you/your family member? Additional question for family member participants: What could have been done for you from the family member perspective?
What does compassionate care mean to you?	What are some of the barriers to compassionate care that you have experienced?

**Table 2:** Sample Codebook.

Code	Definition
Continuity	All references made about examples of continuity, a lack of continuity, or the need for continuity in the continuum of care/transitions across the continuum/how rehabilitation and/or community programs can be maintained over time/long-term follow-up
Costs	All references made about the costs of youth transition or the program costs—financial, human resources/costs to organizations/health care system associated with implementing navigator programs—all references made about the direct and indirect financial costs of implementing such programs/no restrictions on funding these programs/rules for funding programs
Coping/Adaptation	Any description or comment of strategies and methods adopted by the caregiver or youth that are used to manage and deal with emotions and physical health changes related to the condition. For example, when a caregiver or patient mentions using faith or religion to deal with stress and uncertainty. Maintenance/continuum of care/transitions across the continuum/how a program can be maintained over time/long-term follow up. Any coping strategy or changes implemented, that the participant came up with, that allow the participant to cope with the demands of the condition/navigating the health care system or caregiving (excluding help from community services or informal supports, such as help from friends).

**Table 3:** Characteristics of Participants.

Characteristics of Youth (*n* = 11)	
**Sex**	
Female	*n* = 6, 54%
Male	*n* = 5, 46%
Age	μ = 23, σ = 3
Living Environment	
Urban	*n* = 10, 91%
Rural	*n* = 1, 9%
Highest Level of Education	
Obtained Highschool	*n* = 9, 82%
Obtained College/University	*n* = 2, 18%
Ethnicity	
Caucasian	*n* = 8, 73%
Asian	*n* = 3, 27%
Primary Diagnosis	
Cerebral Palsy	*n* = 5, 46%
Acquired Brain Injury	*n* = 3, 27%
Intellectual Disability (various neurodevelopmental disabilities)	*n* = 3, 27%
**Characteristics of Family Caregivers (*n* = 7)**	
Sex	*n* = 7, 100%
Female	
Age	μ = 58, σ = 7
Living Environment	
Urban	*n* = 7, 100%
Highest Level of Education	
Obtained Highschool	*n* = 3, 43%
Obtained College/University	*n* = 4, 57%
Ethnicity	
Caucasian	*n* = 4, 57%
Asian	*n* = 2, 29%
South-East Asian	*n* = 1, 14%
Primary Diagnosis of Care Recipient	
Cerebral Palsy	*n* = 6, 86%
Acquired Brain Injury	*n* = 1, 14%

## Data Availability

The datasets generated and/or analyzed during the current study are not publicly available due to the personal nature of the qualitative data but are available from the corresponding author on reasonable request.
